# Enhanced External Counterpulsation Reduces the Risk of Early Cystatin‐C Rise in STEMI Patients After Primary PCI: A Single‐Center Randomized Controlled Clinical Trial

**DOI:** 10.1155/cdr/4232591

**Published:** 2026-11-15

**Authors:** Chun-Mei Zeng, Guo-Qiang Liu, Hong Lin, Liang Cai, Cai-Zhong Huang, Gui-Qiong Mo, Xin-Jing Zhong

**Affiliations:** ^1^ Department of Cardiolog Yulin First People′s Hospital (The Sixth Affiliated Hospital of Guangxi Medical University) Yulin Guangxi China; ^2^ Guangxi Medical University Nanning Guangxi China gxmu.edu.cn; ^3^ Department of General Practice Yulin First People′s Hospital (The Sixth Affiliated Hospital of Guangxi Medical University) Yulin Guangxi China

**Keywords:** CI-AKI, cystatin C, EECP, primary PCI, STEMI

## Abstract

**Objective:**

To evaluate the effect of enhanced external counterpulsation (EECP) on cystatin‐C in patients with ST‐elevation myocardial infarction (STEMI) following primary percutaneous coronary intervention (primary PCI).

**Methods:**

A total of 436 STEMI patients who underwent primary PCI were enrolled and randomly assigned to the EECP group (*n* = 211) and the control group (*n* = 225). The control group received routine treatment alone, and the EECP group was additionally treated with EECP, which was initiated within 4–12 hours and lasted for up to 72 hours after PCI, with one to two sessions per day and 0.5–1 hour per session. Renal function indicators were detected in all patients before primary PCI, as well as at 24, 72 hours, and 5–7 days after PCI. The differences in renal function parameters, cystatin‐C, and the incidence of contrast‐induced acute kidney injury (CI‐AKI) were compared between the two groups.

**Results:**

Compared with the control group, the EECP group presented significantly lower cystatin‐C at 24, 72 hours, and 5–7 days after PCI (*p* < 0.01), lower serum creatinine (Scr) at 24 hours (*p* = 0.045), and higher estimated glomerular filtration rate (eGFR) (*p* = 0.025). Post‐PCI eGFR decreased markedly in the control group (*p* < 0.001), whereas no significant difference was observed in the EECP group (*p* = 0.361). The linear mixed‐effects model demonstrated a significant between‐group difference in cystatin‐C (*p* < 0.0001), whereas neither the main time effect nor the group‐by‐time interaction reached statistical significance. Patients in the EECP group exhibited a lower risk of increased cystatin‐C (9.5% vs. 4.3%, *χ* ^2^ = 4.359, *p* = 0.037). Nevertheless, there was no statistically significant intergroup difference in the incidence of SCr‐defined CI‐AKI (*p* = 0.584). Firth penalized logistic regression analysis revealed that EECP was an independent protective factor against elevated cystatin‐C (Model 1: OR = 0.45, 95% CI 0.19–0.96, *p* = 0.037; Model 2: OR = 0.44, 95% CI 0.19–0.94, *p* = 0.034).

**Conclusion:**

EECP can reduce cystatin‐C rise after primary PCI in STEMI patients and can represent as a promising intervention targeting biomarkers of early renal injury.

## 1. Introduction

Acute ST‐segment elevation myocardial infarction (STEMI) is characterized by acute onset, rapid disease progression, and high mortality, which seriously threatens the life and health of patients. Primary percutaneous coronary intervention (primary PCI) is the preferred core strategy for the treatment of STEMI patients. It can provide rapid reperfusion of the culprit vessel, effectively reduce myocardial infarct size, and improve cardiac function and long‐term prognosis of patients, thus becoming a key measure for the standardized treatment of STEMI.

However, contrast‐induced acute kidney injury (CI‐AKI), a common complication after PCI, has always been a major concern in clinical practice. Studies have shown that STEMI patients have multiple risk factors including myocardial ischemia and necrosis, impaired cardiac function, and hemodynamic instability. Compared with other patients with coronary heart disease, STEMI patients have a significantly higher incidence of CI‐AKI after PCI [1], which prolongs hospital stay, increases the risk of adverse cardiovascular events, and raises medical economic burden. Hydration, low‐/iso‐osmolar contrast media and strict contrast‐volume restriction constitute the primary clinical measures for CI‐AKI prevention [2, 3]. Nevertheless, for STEMI patients with multiple high‐risk factors, the efficacy of the above measures is still limited, and the risk of CI‐AKI cannot be completely avoided. Accordingly, identifying safer and more effective interventions to reduce post‐PCI CI‐AKI risk may help improve clinical outcomes in STEMI patients.

Serum cystatin‐C (cys‐C) is a sensitive and specific indicator for early renal function injury. Compared with serum creatinine (Scr), it is not affected by age, gender, muscle mass, and other factors and can identify subtle changes in renal function earlier and more accurately [4, 5] and provide a reliable basis for timely clinical intervention. Enhanced external counterpulsation (EECP), a noninvasive circulatory assist therapy, synchronized with the cardiac cycle via electrocardiographic signals, its pneumatic cuffs are inflated sequentially from the distal to proximal extremities during diastole and raises aortic diastolic pressure. The cuffs are then rapidly deflated in systole to reduce peripheral vascular resistance. EECP can improve systemic blood circulation, increase renal perfusion, alleviate ischemia‐reperfusion injury, and exert anti‐inflammatory and antioxidative stress effects, showing potential application value in renal protection. Based on the above evidence, this study is aimed at evaluating the effect of EECP on cys‐C in patients with STEMI following primary PCI and providing new intervention ideas and practical evidence for clinically optimizing perioperative renal protection and reducing the risk of postoperative complications in STEMI patients.

## 2. Materials and Methods

### 2.1. General Data and Study Design

This study adopted a single‐center, randomized parallel‐controlled clinical trial design. A total of 470 consecutive patients with acute STEMI who underwent primary PCI in our hospital were enrolled. The eligible patients were randomly assigned to the EECP group and the control group at a 1:1 ratio.

The control group received conventional treatment alone, and the EECP group was additionally administered EECP therapy. EECP therapy was initiated within 4–12 hours after PCI, continued until 72 hours post‐PCI, with one to two sessions per day and 0.5–1 hour per session.

Renal function parameters were measured in all patients before primary PCI, as well as at 24, 72 hours, and 5–7 days after the procedure. All participants provided written informed consent, and the study protocol was approved by the ethics committee of our hospital (YLSY‐IRB‐SR‐2023007).

### 2.2. Inclusion and Exclusion Criteria

#### 2.2.1. Inclusion Criteria

Patients were eligible for inclusion if they (1) aged ≥ 18 years and < 80 years; (2) met the diagnostic criteria for STEMI, within 12 hours after symptom onset; (3) completed primary PCI; and (4) signed the written informed consent.

#### 2.2.2. Exclusion Criteria

Patients were excluded if they met the following criteria: (1) previous exposure to iodinated contrast agents within 30 days before enrollment; (2) pre‐existing acute kidney injury caused by other definite etiologies; (3) uncontrolled hypertension (blood pressure > 180/110 mmHg); (4) uncontrolled arrhythmias, including tachycardia (heart rate > 120 beats/min), bradycardia (heart rate < 40 beats/min), atrial fibrillation, ventricular tachycardia, etc.; (5) severe congestive heart failure (NYHA Class IV); (6) severe aortic stenosis or aortic regurgitation; (7) aortic, cerebral, or aortic dissection aneurysm; (8) severe peripheral vascular disease, deep venous thrombosis, or phlebitis; (9) bleeding tendency or anticoagulant use with INR > 3.0; (10) severe skin damage, infection, or fracture of the lower extremities; (11) moderate to severe pulmonary hypertension (mean pulmonary artery pressure > 50 mmHg); (12) permanent pacemaker implantation; (13) hemodialysis or peritoneal dialysis; (14) mechanical complications such as cardiac thrombus, cardiac perforation, and papillary muscle rupture; (15) femoral artery puncture access; (16) history of hip replacement, femoral head surgery, or severe lumbar disc herniation; and (17) pregnancy.

### 2.3. Randomization and Blinding

Eligible participants were randomly assigned to the EECP group and the control group via random number tables generated by SPSS 22.0. The random allocation sequence was generated by an independent statistician who was not involved in participant screening, enrollment, or clinical assessment. Subject screening and enrollment were performed by trained and qualified attending physicians, who were blinded to the randomization sequence. The allocation sequence was sealed in sequentially numbered, opaque envelopes; envelopes were opened only after confirming a participant′s eligibility and baseline assessment, which guaranteed allocation concealment before randomization. All personnel responsible for endpoint adjudication remained blinded to group allocation throughout the trial.

### 2.4. Sample Size Calculation

Sample size was calculated using PASS 15 software. Assuming a primary endpoint event rate of 50% in the control group, a relative risk of 0.55, a statistical power of 85%, and an expected dropout rate of 12%, the required total sample size was calculated to be 466. A final sample size of 470 was determined for this study.

### 2.5. Endpoint Events

#### 2.5.1. Primary Endpoint

The incidence of early cys‐C rise, defined as a 25% increase in cys‐C within 24 hours after contrast exposure, was compared with the baseline value.

#### 2.5.2. Secondary Endpoints

1. Differences of cys‐C, Scr, blood urea nitrogen (BUN), estimated glomerular filtration rate (eGFR) at 24, 72 hours, and 5–7 days after primary PCI.
2. The incidence of CI‐AKI diagnosed based on Scr, defined as an increase in Scr of ≥ 0.3 mg/dL (26.5 *μ*mol/L) within 72 hours after contrast exposure, or a 25% elevation relative to baseline Scr level.

### 2.6. Operation Protocol of EECP

The counterpulsation ratio was set at 1:1. The inflation pressure was maintained within 0.020–0.035 MPa, and the sequential inflation duration of air cuffs was 40–50 ms. The ratio of diastolic augmentation wave to systolic wave (D/S) was adjusted to exceed 1.2 as far as possible, and the ratio of diastolic pressure area to systolic pressure area (DP/SP) was controlled at 1.5–2.0. All parameters were further modulated according to individual patient tolerance.

## 3. Statistical Methods

Statistical analyses were performed using SPSS 22.0 and R software. Categorical variables were expressed as *n* (%), and intergroup comparisons were performed using the chi‐square test. Normally distributed continuous variables were presented as mean ± SD; independent‐samples *t*‐test was used for intergroup comparison, and repeated‐measures analysis of variance and linear mixed‐effects model was adopted for multiple intragroup repeated measurements. Firth′s penalized‐likelihood logistic regression analysis was used to explore the influencing factors of cys‐C rise. EECP were enrolled into Model 1, and common risk factors for CI‐AKI were further incorporated into Model 2 based on Model 1. Missing data were handled using complete‐case analysis. A two‐tailed *p* < 0.05 was defined as statistically significant.

## 4. Results

### 4.1. Baseline Characteristics

A total of 470 patients were enrolled in this study. Twenty‐four patients withdrew voluntarily because of a refusal of blood sampling or poor treatment compliance: eight in the control group (six withdrew at the 72‐hour blood collection after randomization, and two withdrew at the 5 to 7‐day blood collection after randomization because they declined blood sampling) and 16 in the EECP group (six withdrew after randomization but before EECP initiation, five withdrew after one to two sessions of EECP, and five refused the 72‐hour blood collection). Another 10 patients were withdrawn from the study for safety reasons owing to heart failure, dizziness, arrhythmia, cerebral infarction, or hypotension; all these withdrawals occurred after randomization and before the start of EECP treatment. Ultimately, 436 patients were included (Figure 1), consisting of 374 males (85.6%) with a mean age of 58.5 ± 10.6 years. Three patients in the EECP group discontinued treatment early because of lower limb pain after 4–5 days of therapy. The proportion of participants completing the full scheduled EECP course was 98.6%, and the mean total EECP treatment duration was 227.5 ± 107.2 minutes. No crossover between groups occurred during the trial. There were no significant between‐group differences in age, body mass index, comorbidities, and preoperative renal function indicators, as well as type and contrast volume (Table 1).

**Figure 1:**
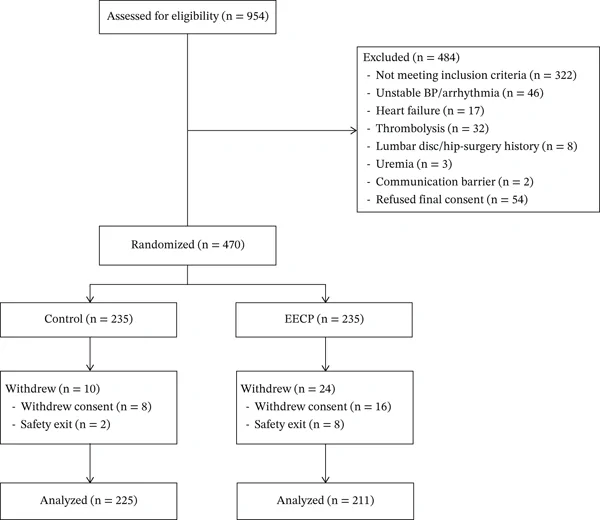
CONSORT diagram.

**Table 1:** Baseline characteristics.

General data	Control group (*n* = 225)	EECP group (*n* = 211)	*χ* ^2^/*t*	*p*
Age (years)	59.0 ± 10.9	57.9 ± 10.3	1.067	0.287
Gender (male [%])	195 (86.7%)	179 (84.8%)	0.300	0.584
BMI (kg/m^2^)	25.0 ± 4.4	24.4 ± 3.1	1.734	0.084
Smoking (*n* [%])	123 (54.7%)	126 (59.7%)	1.133	0.287
Hypertension (*n* [%])	104 (46.2%)	97 (46.0%)	0.003	0.958
T2DM (*n* [%])	39 (17.3%)	35 (16.6%)	0.043	0.836
SBP (mmHg)	137.3 ± 25.8	137.2 ± 22.5	0.053	0.958
DBP (mmHg)	88.2 ± 17.2	88.8 ± 15.8	−0.358	0.721
HR (bpm)	80.6 ± 14.5	80.5 ± 13.6	0.038	0.969
Culprit vessel (*n* [%])			4.079	0.130
LAD	103 (45.8%)	117 (55.5%)		
LCX	29 (12.9%)	22 (10.4%)		
RCA	93 (41.3%)	72 (34.1%)		
No reflow (*n* [%])	1 (0.4%)	1 (0.5%)	0.002	0.964
Hydration (*n* [%])	35 (15.6%)	26 (12.3%)	0.946	0.331
Door‐to‐balloon time (min)	76.9 ± 30.9	75.0 ± 25.0	0.682	0.495
Contrast volume (mL)	102.4 ± 31.8	104.0 ± 30.3	−0.538	0.591
Contrast volume/eGFR ratio	1.4 ± 0.8	1.4 ± 0.7	0.620	0.536
Contrast agent			0.591	0.442
Iso‐osmolar (*n* [%])	213 (94.7%)	196 (92.9%)		
Low osmolar (*n* [%])	12 (5.3%)	15 (7.1%)		
BUN (mmol/L)	5.7 ± 2.3	5.5 ± 1.7	0.840	0.402
Scr (*μ*mol/L)	95.7 ± 28.0	90.8 ± 25.3	1.912	0.057
EGFR (mL/min/1.73m^2^)	81.4 ± 24.4	85.6 ± 24.0	−1.832	0.068
FBG (mmol/L)	6.7 ± 2.4	6.8 ± 2.9	−0.322	0.748
HbA1c (%)	6.9 ± 1.6	6.7 ± 1.8	0.783	0.434
Tc (mmol/L)	5.0 ± 1.1	5.1 ± 1.2	−1.532	0.126
TG (mmol/L)	2.1 ± 1.6	2.0 ± 1.3	0.766	0.444
HDL‐c (mmol/L)	1.0 ± 0.2	1.0 ± 0.3	−1.954	0.051
LDL‐c (mmol/L)	3.2 ± 0.9	3.3 ± 1.0	−1.301	0.194
EF%	51.8 ± 9.2	51.0 ± 9.2	0.852	0.395
FS%	26.8 ± 5.9	26.4 ± 5.8	0.678	0.498
CS	12 (5.3%)	14 (6.6%)	0.329	0.566
CPR	3 (1.3%)	4 (1.9%)	0.218	0.641

Abbreviations: BMI, body mass index; BUN, blood urea nitrogen; CPR, cardiopulmonary resuscitation; CS, cardiogenic shock; DBP, diastolic blood pressure; EF, ejection fraction; eGFR, estimated glomerular filtration rate; FBG, fasting blood glucose; FS, fractional shortening; HDL‐c, high‐density lipoprotein cholesterol; HR, heart rate; LAD, left anterior descending coronary artery; LCX, left circumflex artery; LDL‐c, low‐density lipoprotein cholesterol; RCA, right coronary artery; SBP, systolic blood pressure; Scr, serum creatinine; T2DM, type 2 diabetes mellitus; TC, total cholesterol; TG, triglyceride.

### 4.2. Comparison of Renal Function Indicators Between Two Groups

At 24, 72 hours, and 5–7 days after PCI, serum cys‐C in the EECP group was significantly lower than those in the control group (*p* < 0.01). Meanwhile, the EECP group presented lower Scr (*p* = 0.045) and higher eGFR at 24 hours postoperatively (*p* = 0.025). Post‐PCI eGFR decreased markedly in the control group (*p* < 0.001), whereas no significant difference in eGFR was observed between baseline and post‐PCI values in the EECP group (*p* = 0.361, Table 2 and Figure 2).

**Table 2:** Comparison of renal function indicators between two groups.

Items	Times	Control group (*n* = 225)	EECP group (*n* = 211)	*t*value	*p*
BUN (mmol/L)	Before PCI	5.7 ± 2.3	5.5 ± 1.7	0.840	0.402
	24 hours	5.3 ± 2.3	5.1 ± 1.7	1.068	0.286
	72 hours	5.1 ± 1.9	5.0 ± 1.7	0.377	0.707
	5–7 days	5.3 ± 1.8	5.2 ± 1.9	0.408	0.684
	*χ* ^2^	6.460	5.144		
	*p*	0.002	0.003		

Scr (*μ*mol/L)	Before PCI	95.7 ± 28.0	90.8 ± 25.3	1.912	0.057
	24 hours	94.9 ± 26.6	90.0 ± 24.2	2.011	0.045
	72 hours	97.2 ± 24.2	94.4 ± 27.4	1.109	0.268
	5–7 days	99.1 ± 25.6	96.1 ± 30.0	1.051	0.294
	*χ* ^2^	5.755	12.013		
	*p*	0.003	< 0.001		

EGFR (mL/min/1.73m^2^)	Before PCI	81.4 ± 24.4	85.6 ± 24.0	−1.832	0.068
	24 hours	81.3 ± 22.4	86.3 ± 24.1	−2.256	0.025
	72 hours	78.3 ± 20.8	80.9 ± 21.1	−1.300	0.194
	5–7 days	76.4 ± 20.8	80.6 ± 22.6	−1.882	0.061
	*χ* ^2^	12.117	0.841		
	*p*	< 0.001	0.361		

Cystatin‐C (mg/L)	Before PCI	1.1 ± 0.4	1.1 ± 0.3	1.828	0.068
	24 hours	1.2 ± 0.3	1.1 ± 0.3	3.604	< 0.001
	72 hours	1.2 ± 0.3	1.1 ± 0.3	3.573	< 0.001
	5–7 days	1.2 ± 0.3	1.1 ± 0.3	3.192	0.002
	*χ* ^2^	1.056	1.009		
	*p*	0.358	0.379		

**Figure 2:**
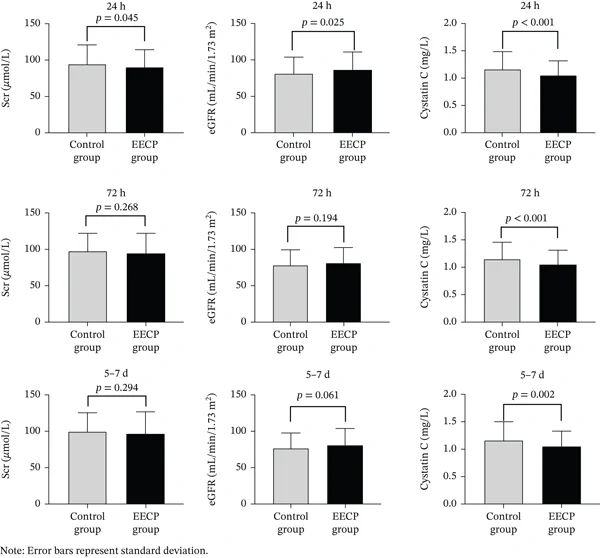
Comparison of renal function after PCI between the two groups.

Linear mixed‐effects models were applied to adjust for baseline renal function indices. (1) Cys‐C:linear mixed‐effects models (random intercept, REML) adjusted for baseline cys‐C were used to analyze serial cys‐C levels. The group‐by‐time interaction was nonsignificant (*F* = 0.037, *d* *f* = 2, 782, *p* = 0.9636), indicating parallel temporal trajectories between groups. There was a significant main effect of group (*F* = 44.598, *d* *f* = 1, 429, *p* < 0.0001): EECP was associated with persistently lower cys‐C (*β* = −0.0614, 95% CI −0.0973 to −0.0255, *p* = 0.0008). The main effect of time was not significant (*F* = 0.131, *d* *f* = 2, 782, *p* = 0.8769). Baseline cys‐C strongly predicted subsequent values (*β* = 0.6688, 95% CI 0.6237–0.7140, *p* < 0.0001). Bonferroni‐corrected pairwise comparisons showed significantly lower cys‐C in the EECP group at all time points: baseline (difference 0.0614, *p* = 0.0008), 72 hours (difference 0.0638, *p* = 0.0006), and 5–7 days post‐PCI (difference 0.0662, *p* = 0.0006). Adjusted mean cys‐C values were 1.14, 1.14, and 1.14 in controls and 1.08, 1.08, and 1.07 in the EECP group at the three time points, respectively. (2) eGFR: The group‐by‐time interaction was not significant (*F* = 0.944, *d* *f* = 2, 798, *p* = 0.3894). No significant main effects of group (*F* = 2.127, *d* *f* = 1, 429, *p* = 0.1455) or time (*F* = 0.866, *d* *f* = 2, 798, *p* = 0.4212) were observed. Baseline eGFR was a strong independent predictor of subsequent eGFR (*β* = 0.9268, 95% CI 0.5577–1.2959, *p* < 0.0001). Bonferroni‐corrected pairwise comparisons showed no statistically significant between‐group differences at baseline (difference −1.196, *p* = 0.9382), 72 hours (difference −27.239, *p* = 0.0810), or 5–7 days post‐PCI (difference −0.692, *p* = 0.9663). The adjusted mean eGFR values for controls versus the EECP group were 82.9 vs. 84.1 at baseline, 79.8 vs. 107.0 at 72 hours, and 78.3 vs. 79.0 at 5–7 days. (3) Scr: The group‐by‐time interaction was not significant (*F* = 0.941, *d* *f* = 2, 799, *p* = 0.3906). There was a significant main effect of group (*F* = 9.332, *d* *f* = 1, 429, *p* = 0.0024) and a significant main effect of time (*F* = 26.096, *d* *f* = 2, 799, *p* < 0.0001). Scr increased significantly over time after PCI (time2: *β* = 2.6016, 95% CI 0.8082–4.3950, *p* = 0.0045; time3: *β* = 3.8029, 95% CI 1.9248–5.6809, *p* = 0.0001). Baseline Scr strongly predicted subsequent Scr values (*β* = 0.7907, 95% CI 0.7441–0.8374, *p* < 0.0001). Bonferroni‐corrected pairwise comparisons showed no significant between‐group differences at any time point: baseline (difference 1.111, *p* = 0.4524), 72 hours (difference −0.387, *p* = 0.7946), and 5–7 days post‐PCI (difference −0.547, *p* = 0.7205). Adjusted mean Scr values for controls versus the EECP group were 93.2 vs. 92.1 at baseline, 95.8 vs. 96.2 at 72 hours, and 97.0 vs. 97.6 at 5–7 days. Plots illustrating temporal changes in adjusted renal function indices are presented in Figure 3.

**Figure 3:**
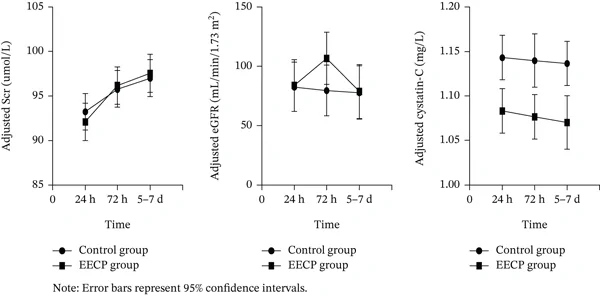
Trends of adjusted renal function indicators between the two groups.

### 4.3. Comparison of Endpoint Events Between Two Groups

Patients in the EECP group had a lower risk of early cys‐C elevation (9.5% vs. 4.3%, *χ* ^2^ = 4.359, *p* = 0.037, RR = 0.457 (95% CI: 0.217–0.955), ARR = 0.051 (95% CI: −0.001 to 0.103), and NNT = 19.73). Nevertheless, there was no significant between‐group difference in the risk of Scr‐defined CI‐AKI (p = 0.584, Table 3).

**Table 3:** Comparison of endpoint events between the two groups.

Items	Control group (*n* = 225)	EECP group (*n* = 211)	*χ* ^2^value	*p*
Cystatin‐C early rise (*n* [%])	21 (9.5%)	9 (4.3%)	4.359	0.037
CI‐AKI based on Scr (*n* [%])	18 (8.0%)	20 (9.5%)	0.299	0.584

Abbreviations: CI‐AKI, contrast‐induced acute kidney injury; Scr, serum creatinine.

### 4.4. Regression Analysis

Early cys‐c rise was set as the dependent variable. Model 1 included EECP as the independent variable, whereas Model 2 added well‐established risk factors for CI‐AKI to Model 1.

The results revealed that EECP was an independent protective factor against early cys‐c rise (Model 1: OR = 0.45, 95% CI 0.19–0.96, *p* = 0.037; Model 2: OR = 0.44, 95% CI 0.19–0.94, *p* = 0.034; Table 4).

**Table 4:** Firth regression.

Items	*p*	OR	95% CI	
			Lower limit	Upper limit
Model 1				
EECP	0.037	0.45	0.19	0.96
Model 2				
EECP	0.034	0.44	0.19	0.94
Gender	0.153	0.50	0.21	1.32
T2DM	0.426	1.45	0.56	3.38
Hypertension	0.647	0.84	0.39	1.76
Contrast volume	0.577	1.00	0.99	1.01

Abbreviations: Model 1, univariable analysis; Model 2, multivariable analysis.

### 4.5. Safety Analysis

The incidence of adverse events was analyzed according to the randomization allocation. No statistically significant difference was observed between the two groups (*p* > 0.05), as shown in Table 5.

**Table 5:** Adverse events.

	Control group (*n* = 235)	EECP group (*n* = 235)	*χ* ^2^value	*p*
Adverse events	19 (8.1%)	23 (9.8%)	0.418	0.518
Heart failure	6 (2.6%)	3 (1.3%)	1.039	0.308
Angina pectoris	5 (2.1%)	7 (3.0%)	0.342	0.559
Arrhythmia	4 (1.7%)	4 (1.7%)	0.000	1.000
Hypotension	1 (0.4%)	3 (1.3%)	1.055	0.304
Death	1 (0.4%)	0 (0%)	1.388	0.239
Others	2 (0.9%)	6 (2.6%)	2.128	0.145

## 5. Discussion

This study adopted cys‐C as an early sensitive biomarker for renal injury to explore the protective effect of EECP on early cys‐c rise in patients with STEMI undergoing primary PCI. Despite several limitations existing in this research, the following conclusions were drawn. Firstly, EECP can ameliorate postoperative renal function parameters in STEMI patients after primary PCI. Secondly, EECP reduces the risk of early cys‐c rise by 66.3%. Thirdly, EECP fails to decrease the risk of CI‐AKI diagnosed according to Scr criteria.

Previous studies on CI‐AKI mostly adopted Scr as the diagnostic indicator. In 2012, the Kidney Disease: Improving Global Outcomes (KDIGO) guidelines redefined CI‐AKI, which was defined as a ≥ 1.5‐fold increase in Scr from baseline within 7 days after contrast exposure, an elevation of Scr ≥ 0.3 mg/dL (26.5 *μ*mol/L) within 48 hours, or oliguria with urine output less than 0.5 mL·kg^−1^·h^−1^ persisting for more than 6 hours [6]. Scr is greatly affected by age, muscle mass, nutritional status and fluid volume, and its elevation lags behind the occurrence of renal impairment. In contrast, cys‐C is barely influenced by nonrenal factors. It can sensitively reflect early glomerular filtration dysfunction and renal tubular injury, serving as a more sensitive biomarker for CI‐AKI diagnosis [7, 8]. Hence, it is more suitable for early screening of perioperative CI‐AKI in patients undergoing PCI, and relevant research conclusions possess better clinical timeliness.

The exact pathogenesis of CI‐AKI has not been fully elucidated. Direct cytotoxicity induced by contrast medium (CM), intrarenal hemodynamic alterations, and oxidative stress are recognized as the predominant pathophysiological mechanisms of CI‐AKI. These three mechanisms interact and exacerbate each other, thereby forming a vicious cycle [9–11].

STEMI patients frequently develop low cardiac output, hypoperfusion, reperfusion injury, and stress‐induced hyperglycemia [12, 13] and are at high risk of CI‐AKI. Previous studies have demonstrated that the incidence of CI‐AKI ranges from 9.1% to 16% in STEMI patients undergoing primary PCI according to Scr‐based diagnostic criteria [14–16]. Although the popularization of iso‐osmolar and low‐osmolar contrast agents has reduced its risk, the incidence still remains at 7.4%–11.9% [17, 18]. The CI‐AKI incidence defined by Scr in the present study was consistent with those reported in previous literature. Cys‐C is superior to serum creatinine in the early diagnosis of CI‐AKI, yet unified diagnostic criteria have not been established. Most studies adopt a 25% increase in cys‐C level within 24 hours after contrast exposure relative to baseline as the diagnostic cutoff. In Michal′s research, the incidence of cys‐C‐defined CI‐AKI reached 70.4% after primary PCI in STEMI patients [19], which was markedly higher than that observed in our study (9.5% in the control group and 4.3% in the EECP group). This discrepancy may be attributed to the relatively younger age of enrolled patients (58.5 ± 10.6 years) and the exclusive application of iso‐osmolar or low‐osmolar contrast agents in this trial.

EECP improves visceral organ function via electrocardiogram‐triggered compression on buttocks and lower extremities. It has been widely applied in the management of angina pectoris, cerebrovascular diseases, and heart failure and can enhance exercise tolerance and quality of life in patients [20, 21] with favorable tolerability and safety in most populations [22]. Werner et al. [23] verified that EECP could elevate stroke volume, mean arterial pressure, atrial natriuretic peptide (ANP) level, glomerular filtration and urine flow rate, reduce plasma renin concentration, and facilitate renal excretion and diuresis in healthy subjects. Rua et al. confirmed that EECP could improve long‐term renal function in patients with cardiac diseases. After EECP intervention, cys‐C level decreased significantly from 1.00 (0.78–1.31) to 0.94 mg/L (0.77–1.27) (*p* < 0.001), whereas eGFR increased from 70.47 (43.88–89.41) to 76.27 mL/min/1.73 m^2^ (49.02–91.46) (*p* = 0.006). Subgroup analysis further revealed a notable decline of cys‐C in patients with baseline eGFR < 60 mL/min/1.73 m^2^ (*p* < 0.01) [24].

In recent years, growing researchers have focused on the protective effect of EECP against contrast‐induced renal injury, indicating that EECP provides a novel strategy for the prevention and treatment of CI‐AKI [25, 26]. Our previous studies found that EECP could reduce Scr level (151.9 ± 44.7 vs. 144.5 ± 48.3 * μ*mol/L, *p* < 0.001), elevate eGFR (43.6 ± 11.4 vs. 47.1 ± 13.9 mL/min/1.73 m^2^, p < 0.001) and lower CI‐AKI risk (1.1% vs. 6.0%, *p* = 0.019) in CKD patients after coronary angiography or PCI [27], and similar outcomes were observed in diabetic patients complicated with CKD [28]. Zhang′s study enrolled patients undergoing diagnostic contrast‐enhanced computed tomography. The primary endpoint was a 10% elevation in cys‐C, whereas the incidence of SCr‐defined CI‐AKI served as the secondary endpoint. That study demonstrated that EECP could reduce the rate of cys‐C elevation and the incidence of CI‐AKI, as well as improve contrast‐media clearance [29]. The present study is similar to Zhang′s study; however, there are differences in the study population, route of contrast‐medium administration, and EECP protocol. Moreover, we adopted a stricter criterion for cys‐C elevation, which allows a more accurate evaluation of renal function changes in STEMI patients after primary PCI. Unlike the findings reported by Zhang, no intergroup difference in the incidence of SCr‐defined CI‐AKI was observed in our study.

EECP exerts protective effects against CI‐AKI through multiple mechanisms. Firstly, EECP can increase renal blood flow by 19% [30], thereby ameliorating renal perfusion and alleviating renal ischemia and hypoxia. Secondly, EECP elevates ANP levels (11 ± 4 vs. 16 ± 5 ng/L, *p* < 0.01) and improves urine flow rate (1.8 ± 1.5 vs. 2.8 ± 1.9 mL/min, *p* < 0.05). It enhances renal perfusion, facilitates renal excretion, and improves diuretic effect [23], which accelerates CM clearance [25] and reduces renal retention of contrast agents. Thirdly, fluid shear stress induced by EECP activates the Nrf2 pathway to upregulate the expression and maintain the function of multidrug and toxic compound extrusion transporter (MATE), thus mitigating the cytotoxicity of CM [31, 32]. Meanwhile, EECP promotes nitric oxide production and improves endothelial function, which relieves CM‐induced oxidative stress [33]. Fourthly, via diastolic augmentation and systolic unloading, EECP elevates cardiac output, coronary blood flow, and myocardial perfusion in STEMI patients [34, 35]; reduces myocardial infarct size; optimizes systemic organ perfusion; and further lowers the risk of CI‐AKI.

Unfortunately, we failed to observe a reduction in the risk of CI‐AKI diagnosed according to Scr‐based criteria in the present study. Although isolated improvement in biomarkers does not translate into benefits for clinical outcomes and EECP‐associated treatment may raise medical costs to some extent, our findings offer a novel research direction for the prevention of CI‐AKI. Meanwhile, the clinical advantages of EECP within cardiology are well‐established, including enhanced coronary blood supply, relieved angina symptoms, and improved quality of life in patients with heart failure [36, 37].

Cys‐C, the primary endpoint of this study, had missing values at all time points, mainly attributable to medical insurance constraints, unsuccessful blood sampling, and damaged specimens unavailable for testing. The proportions of missing data were 1.61% before PCI, 0.92% at 24 hours post‐PCI, 3.9% at 72 hours post‐PCI, and 14.9% at 5–7 days post‐PCI. The adoption of complete‐case analysis for missing data may reduce the sample size and statistical power, introduce attrition bias, and limit the generalizability of the results.

Postrandomization withdrawals create a risk of attrition bias in this trial. The 34 excluded patients were unevenly distributed between groups, with more voluntary dropouts in the EECP arm. Patients who withdrew may have differed in cys‐C changes and cardiovascular risk from those retained in the analysis, which could bias the estimated treatment effect. We did not perform an intention‐to‐treat analysis because the primary endpoint required timed cys‐C measurements, and missing biomarker data could not be reliably imputed. All dropout reasons and timings are documented in the CONSORT flowchart. This limitation should be noted when interpreting our findings.

Several limitations exist in the present study. First, because of financial constraints, other sensitive AKI biomarkers such as neutrophil gelatinase‐associated lipocalin (NGAL) were not detected. Second, urinary contrast agent excretion rate was not measured, and the specific molecular mechanisms by which EECP reduces CI‐AKI risk were not further verified. Third, we did not further explore whether elevated cys‐C levels correlate with clinical outcomes, including all‐cause mortality, persistent renal impairment, chronic kidney disease progression, and dialysis dependence. Fourth, because the incidence of the primary endpoint was substantially lower than estimated, the study may be underpowered. Larger sample investigations are warranted in future research. Fifth, relatively strict exclusion criteria were applied; male patients accounted for 85.6% of participants. Accordingly, our enrolled cohort represented a screened, lower risk STEMI population. Caution should be taken when extrapolating the present findings to an unselected real‐world STEMI patient population. Sixth, D/S and DP/SP waveform parameters were not recorded, so we could not quantify the intensity of EECP or verify whether sufficient hemodynamic loading was achieved during each treatment session.

## 6. Conclusion

EECP can reduce cys‐C rise after primary PCI in STEMI patients and represents a promising intervention targeting biomarkers of early renal injury.

## Author Contributions

Conception and design of the research: Chun‐Mei Zeng. Acquisition of data: Chun‐Mei Zeng, Guo‐Qiang Liu, Liang Cai, Xin‐Jing Zhong, Cai‐Zhong Huang, and Gui‐Qiong Mo. Analysis and interpretation of the data: Chun‐Mei Zeng, Guo‐Qiang Liu, Hong Lin, Liang Cai, Xin‐Jing Zhong, Cai‐Zhong Huang, and Gui‐Qiong Mo. Assessment of endpoint events: Hong Lin. Statistical analysis: Guo‐Qiang Liu and Chun‐Mei Zeng. Obtaining financing: Chun‐Mei Zeng. Writing of the manuscript: Chun‐Mei Zeng and Guo‐Qiang Liu. Critical revision of the manuscript for intellectual content: Chun‐Mei Zeng.

## Funding

This study was supported by the Guangxi Health Care Commission Project: Efficacy of EECP for Preventing CI‐AKI Post–Primary PCI in STEMI Patients (Z‐K20231764).

## Disclosure

All authors read and approved the final draft.

## Ethics Statement

This study was conducted with approval from the Ethics Committee of Yulin First People′s Hospital (No.YLSY‐IRB‐SR‐2023007) and registered on the Chinese Clinical Trial Register website (http://www.chictr.org.cn, ChiCTR2300069005). This study was conducted in accordance with the Declaration of Helsinki.

## Consent

Written informed consent was obtained from all participants.

## Conflicts of Interest

None of the authors have a conflict of interest to disclose.

## Data Availability

The data that support the findings of this study are available on request from the corresponding author. The data are not publicly available due to privacy or ethical restrictions.
